# A double guidance mechanism, nitroaniline based microstructured optical fiber

**DOI:** 10.1038/s41598-018-33855-4

**Published:** 2018-10-22

**Authors:** Georgios Violakis, Stavros Pissadakis

**Affiliations:** Foundation for Research and Technology-Hellas (FORTH), Institute of Electronic Structure and Laser (IESL), N. Plastira 100, 70013 Heraklion, Greece

## Abstract

A new type of all-solid, photonic bandgap fiber exhibiting a wavelength dependent guidance mechanism and second harmonic generation capabilities is presented. A silica glass microstructured optical fiber was infiltrated with 2-methyl 4-nitroaniline for creating the composite material optical fiber. This optical fiber was characterized over a broad wavelength range, revealing that a transition from photonic bandgap guidance to modified total internal reflection propagation occurs from short to longer wavelengths, attributed to the dispersion characteristics of the low Abbe number nitroaniline. Annealing post-processing was used for tuning the morphology of the solidified nitroaniline inside the capillaries of the silica glass microstructured optical fiber which increased the extinction ratio of the transmission bandgaps. This composite material optical fiber also exhibits second harmonic generation capabilities under 1064 nm laser excitation, with conversion characteristics dependent upon the packing of the nitroaniline inside the optical fiber capillaries. As the pump and generated light fall into different guidance regimes of the optical fiber, such a device could be potentially used as an all optical gate or light conversion device.

## Introduction

Organic crystals have been extensively researched due to their optical polarizability properties, rendering them useful in wavelength conversion, optical switching and limiting^1^. An organic crystal that has attracted early attention for its high second harmonic generation (SHG) efficiency and large molecular first order hyper-polarizability is the 2-methyl 4-nitroanline (ΜΝΑ)^2,3^. Compared to highly non-linear inorganic crystals such as LiNbO_3_, the SHG coefficient d_11_ for MNA is ~40 times larger^2,4^. Integration of MNA as thin film overlayer in grating couplers and waveguides has shown that this material can be potentially used for light gating^5^. Studies of electrospun grown MNA crystals showed the importance of molecular orientation and crystal arrangement for realizing, highly non-linear optical devices^6^, highlighting also the relation between MNA crystal size and SHG intensity^7^. Theoretical calculations of the SHG performance of an MNA-core cylindrical waveguide have been performed in the past^8^ and a loss-hindered device has been presented^9^. In general, the high absorption and scattering loss of MNA-cored waveguides have limited their practical implementation. These obstacles can be overcome by the combination of MNA crystals in an optical system with controlled and wavelength dependent modal interaction between the probe light and the MNA material. Microstructured optical fibers (MOFs), comprised of micrometric diameter longitudinal capillaries on a multi-ring arrangement^10^ can provide controlled modal interaction of the light with the infiltrated MNA material^11,12^ and prompt preferential crystallization orientation. Accordingly, by tuning the refractive index of the infiltrated materials inside the MOF capillaries, different modal propagation effects or guidance mechanisms can be triggered. There has been considerable work where MOF capillaries are filled with inorganic glasses^13,14^, crystals^15^, polymer dispersed liquid crystals^16^, liquids^17^ or polymers^18^ inducing modified total internal reflection (MTIR) or anti-resonant guiding mechanisms.

In this manuscript, MNA is infiltrated from melt inside an endlessly single mode silica glass MOF, for realizing an all-solid optical fiber which supports more than one guiding mechanisms with respect to the propagating light wavelength. Namely, the photonic bandgap guidance (PBG) occurring at short wavelengths transforms to a MTIR propagation at longer wavelengths, with an intermediate high loss spectral band. This composite fiber is also pumped with 1064 nm pulsed laser light and the generation of second harmonic at 532 nm from the high refractive index MNA strands is also demonstrated.

## Results and Discussion

### Characterization

**Figure 1:**
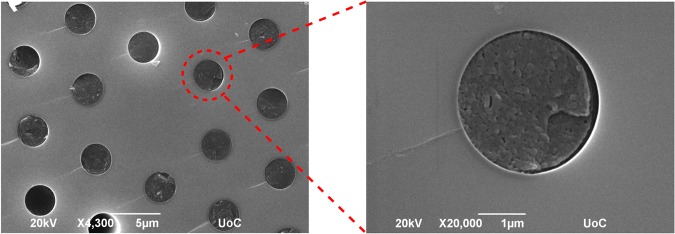
Scanning Electron Microscopy image of the MNA infiltrated fiber. (Left) Area surrounding the optical fiber core. (Right) MNA-filled inner-ring capillary. Infiltration time: 24 h.

The composite MNA infiltrated optical fibers were fabricated by immersion of LMA-10 optical fiber in a MNA melt and filled by capillary forces. These composite optical fibers were examined with both optical and electronic microscopy to examine infiltration lengths, filling quality and structural growth of the MNA inside the micrometric-sized capillaries. A typical scanning electron microscopy (SEM) image of an ‘as-fabricated’, MNA-infiltrated MOF (MNA-LMA) is presented in Fig. 1, with the conditions presented in Methods. SEM images suggest solidification of MNA into a polycrystalline matrix^19^. There are occurrences of voids or partially filled MOF capillaries probably as a result of their full or partial blockage.

**Figure 2:**
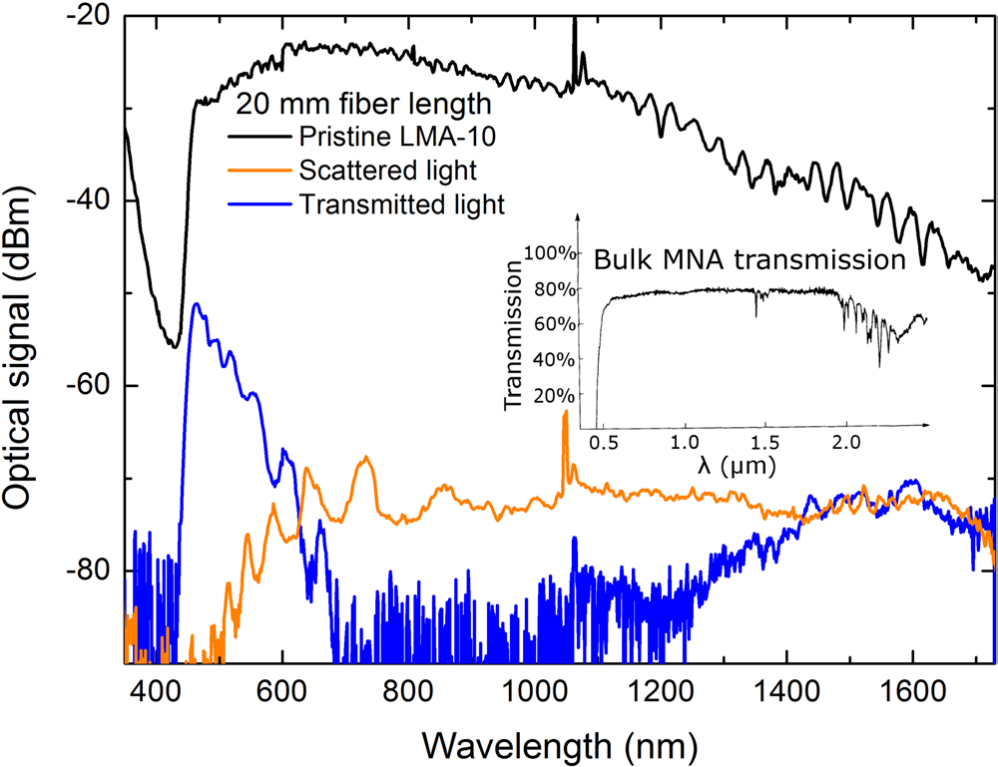
Transmission spectra of 20 mm long optical fibers: pristine LMA-10 (black line) and MNA-filled LMA-10 (blue line). Scattered light spectrum from the side of the MNA-LMA fiber (orange line). Graph inset: MNA transmittance, modified from^2^.

Following fabrication, MNA-infiltrated MOFs were endface cleaved and spectrally characterized. The result of filling LMA-10 with MNA is the substantial alteration of the transmittance spectrum of the fiber compared to that of the pristine (air-filled capillaries) LMA-10. The transmission (blue) and out-cladding scattered light spectra (orange line) of a 20 mm long MNA-LMA fiber are presented in Fig. 2 alongside the transmission spectrum of a pristine LMA-10 fiber of equal length (black line).

Examination of the MNA-LMA fiber transmission spectrum reveals the formation of distinct spectral features at a wavelength range between 470 nm and ~750 nm, a high loss transmittance region ranging from ~800 nm to 1200 nm and a gradual transmittance increase from 1200 nm up to the spectrum analyzer’s readout range (1750 nm). MNA absorption spectrum is flat from a wavelength range of ~550 nm to ~1900 nm^2^ (see inset graph of Fig. 2) and cannot justify the high loss gap observed from 800 nm to 1200 nm. In the visible band, shallow spectral modulations of the transmission amplitude appear, which depend upon the homogeneity of the MNA filling of the capillaries, especially those in direct proximity to the fiber core^14^. The spectral notches observed in the short wavelength transmission band (shorter than 800 nm) are attributed to PBG guidance, and are further confirmed by light scattering measurements from the composite optical fiber cladding, where complementary peaks of anti-guidance clearly appear (see orange solid line in Fig. 2).

### Refractive index calculations

**Figure 3:**
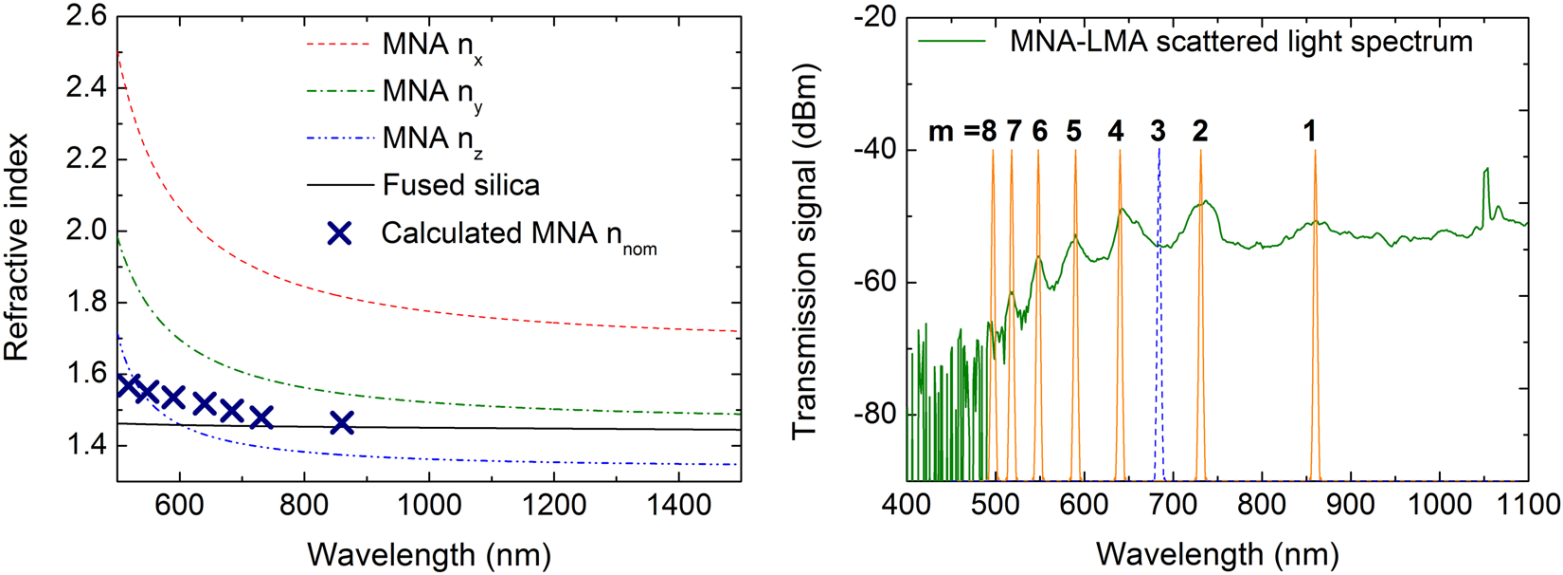
(Left) RI dispersion curves calculated for MNA and fused silica (solid and dashed lines) using Sellmeier coefficients from^20^ and^26^, respectively. Calculated nominal MNA RI n_nom_ using equation (1) (‘x’ marks). (Right) Location of composite optical fiber bandgaps from MNA-LMA scattering spectrum and requirement for a sequential λ_m_ order. Peak at modal position m = 3 is plotted as a blue dashed line, as it appears mostly in the transmission spectrum of other MNA-LMA samples (see Figs 4 and 5).

Important factors dominating the spectral transmission of this specialty optical fiber are both the orientation of the MNA microcrystals inside the MOF capillaries, and their corresponding volume packing. MNA is a low Abbe number (V_d_ < 3), biaxial crystal, with two out of three principal axes exhibiting refractive indices larger than silica for all investigated wavelengths (n_x_ and n_y_), while the n_z_ rests lower than silica for wavelengths longer than ~600 nm^20^ (see Fig. 3). From the experimental data available herein, the exact orientation of the MNA microcrystals inside the MOF cannot be readily determined, or even correlated with the refractive index ellipsoid^21^ of this organic material. However, a basic insight of the refractive index dispersion of the MNA inside the LMA-10 capillaries can be gained from the short wavelength PBG spectra. In particular, from the spectral locations of the photonic bandgaps appearing in the visible (to NIR) out-cladding scattering spectrum, a nominal refractive index of the MNA material was calculated for different wavelengths using the anti-resonant reflecting optical waveguide (ARROW) model^22^. This nominal refractive index *n*_*nom*_ is a statistical quantity denoting the multiple randomly oriented MNA micro-crystals that sum up along the fiber length. A similar interpretation is also followed in ceramics and polycrystalline polymers^23–25^. According to Litchinitser *et al*.^22^, the transmission dips (*λ*_*m*_) of a photonic bandgap guidance optical fiber can be calculated by solely knowing the capillary diameter (*d*) and infiltrated material RI (*n*_*cap*_), as well as, the host fiber material RI (*n*_*core*_), according to equation:

1 $${\lambda }_{m}=\frac{2d\sqrt{{n}_{cap}^{2}-{n}_{core}^{2}}}{m+1/2}\,m=1,2,$$

Knowing the bandgap locations, one can solve equation (1) as a function of n_cap_ and acquire the RI at the specific wavelengths where bandgap formation is observed. The RI of fused silica (n_core_) can be calculated by using the coefficients in^26^ and the diameter of the LMA-10 capillaries *d* (see Methods). For the calculation of the MNA nominal refractive index through equation (1), bandgaps should appear in sequential order, starting from longer and moving towards shorter wavelengths. Taking into consideration the above, the resulting RI from equation (1) is plotted against wavelength in the left part of Fig. 3 as ‘x’ marks, along with the dispersion curve of monocrystalline MNA (according to^20^) and fused silica (using data from^26^). The calculated nominal refractive index dispersion is expected to reside between the highest (n_x_) and lowest (n_z_) MNA refractive index and follow a similar shape. However, the calculations reveal nominal MNA RI values lower than those of the n_z_ axis at wavelengths below ~550 nm with a less steep rise towards this spectral region. This feature is directly associated with the calculations requirement of sequential bandgap appearance. Relaxation of this rule and reassignment of bandgaps appearing at short wavelengths to higher m orders would yield higher nominal MNA RI values for short wavelengths, exhibiting a dispersion similar to that of MNA^20^. The last would imply the existence of full or partial bandgaps in the composite optical fiber; however, not being experimentally observed. Another possible source for the shape of the calculated nominal MNA RI can be the incomplete filling of the MOF capillaries and the existence of voids between the micro-crystals that can modify the dispersion shape.

**Figure 4:**
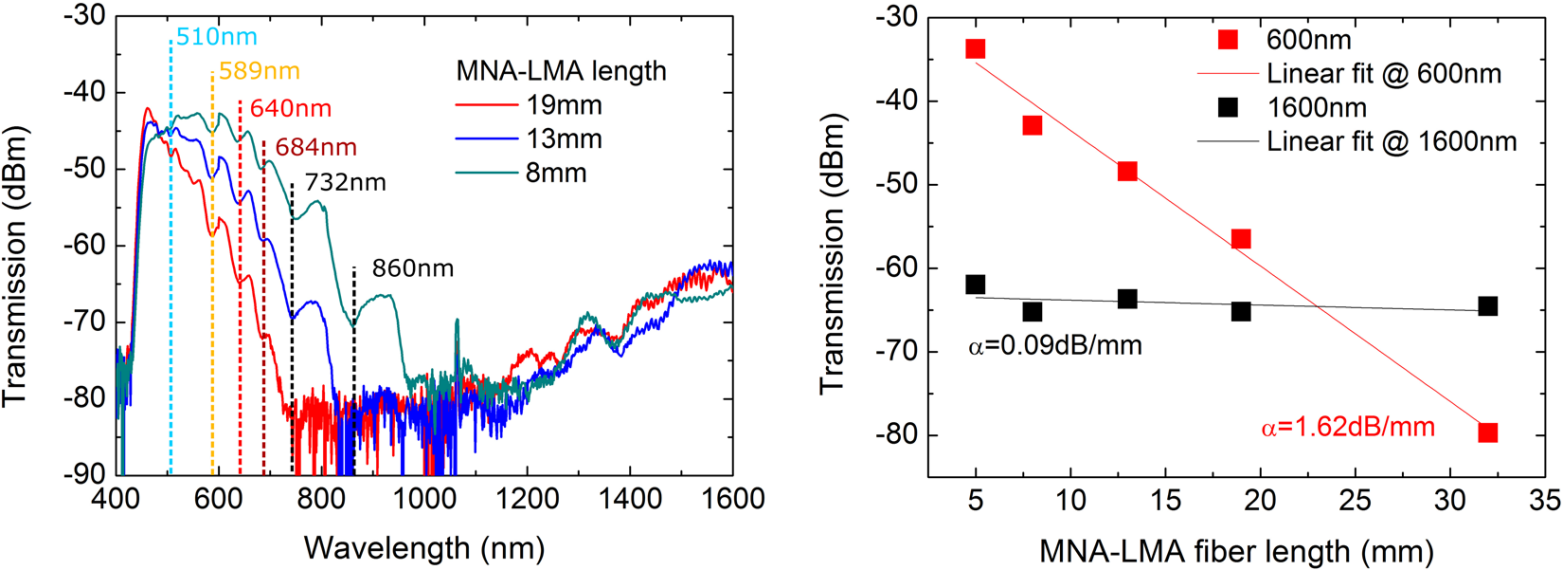
Left: Transmission spectra of a cut-back MNA-LMA sample along with notation of predicted bandgap location using ARROW model^22^. Right: Losses as a function of optical fiber length for a wavelength of 600 nm and 1600 nm.

**Figure 5:**
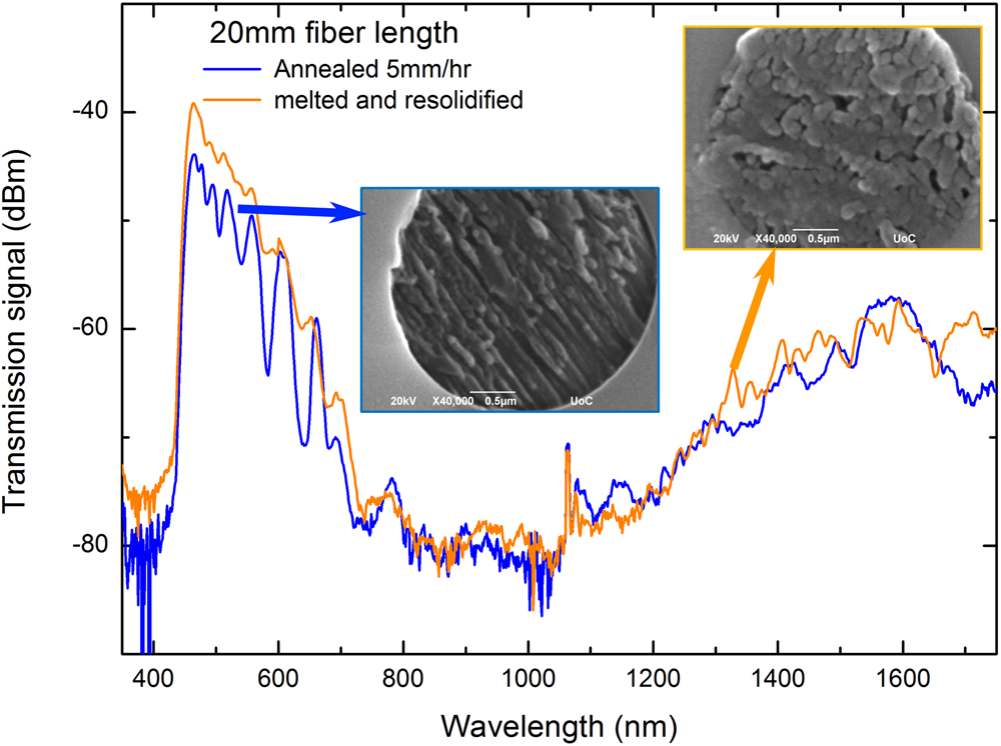
Transmission spectra of an annealed and a rapidly cooled (after melting MNA) optical fiber along with their respective capillary SEM images.

In the right part of Fig. 3, the bandgap locations are plotted against the scattered light spectrum of an MNA-LMA fiber. The 684 nm peak at modal position m = 3 has been plotted as a dashed line, because it was not observed in the scattered light spectrum of the specific sample, but systematically appears in transmission spectra of other MNA-LMA samples that are presented in the following sections of this work (Figs 4 and 5). Attempts to shift the first anti-resonant mode m = 1 to longer wavelengths and perform peak allocation calculations, did not match the experimentally obtained spectra.

The proximity of the calculated nominal MNA RI to the MNA n_z_ RI shows the alignment of the specific optical axis of the material with the propagation k-vector of the guided mode, thus, a preferential arrangement of MNA crystallographic z axis along the MOF capillaries is assumed. Similar behavior to the above has been reported before, where molecules similar to MNA (that of meta-nitroaniline) crystallize with their long crystallographic axis aligned to the waveguide axis^27^.

Assuming the nominal refractive index dispersion curve of Fig. 3 evaluated from the PBG peak position, three guiding zones can be defined. Initially, a photonic bandgap guidance mechanism is manifested in the visible spectrum up to ~850 nm, where the average MNA refractive index is higher than that of silica, a MTIR guidance located in the region above ~1100 nm where the average MNA RI is lower than that of silica, with an intermediate high loss region where the MNA RI is close to that of silica, rendering the composite optical fiber a large light pipe.

Supplementary evidence of two distinct guidance mechanisms is obtained by examination of the transmission spectra of an MNA-LMA sample gradually cut-back from 32 mm to 5 mm. As presented in the left part of Fig. 4 (spectra of 19 mm to 8 mm samples), losses in the visible part of the spectrum are significantly increasing with fiber length while they remain practically constant in the NIR region. Attenuation in the later zone was calculated using Beer-Lambert’s law to be 0.09 dB/mm and reaches a value of 1.62 dB/mm in the visible (right part of Fig. 4). A plausible explanation of this behavior is the existence of partial bandgaps along the propagation direction that add up with fiber length. MTIR guidance is less affected by incomplete packing of the MNA and thus no major loss increase is observed in this zone with increasing sample length.

### Annealing

Crystallization of MNA plays a major role in the transmission properties of the composite fiber. Post-processing of the fibers by means of thermal annealing was performed in order to achieve a better crystallization of the confined MNA material. The MNA based optical fibers are annealed and retracted from the temperature gradient in the oven using the protocol described in Methods and pulling speeds similar to those reported in the literature^20,28^.

The annealing results of a 20 mm long MNA-LMA fiber are presented in Fig. 5 and compared to the transmission spectrum of a fiber overheated and rapidly cooled. The solidified MNA in the two optical fibers possesses a clearly different microscopic arrangement as seen from the SEM images of an inner ring capillary from both fibers in the inset of Fig. 5. As a result, the strengths of the formed spectral bandgaps in the annealed fiber are greater than that of the rapidly re-solidified fiber. For instance, the bandgap formed at 640 nm grows from 3.3 dB to 11.1 dB for the annealed fiber and previously weak or undetectable bandgaps can now be observed, as the bandgap at 542 nm that was previously not formed. The pronounced bandgap strength in the annealed fiber with the improved structure in the capillary is indicative of the effect of crystal orientation and void filling in the performance of the composite fiber. The rapidly cooled fiber has bandgaps that are still located in the same positions but much weaker. This could be the result of partial bandgap formation in the x and y axes of the fiber cross-section at the expense of bandgap strength in the z (propagation) axis due to a more random crystal orientation in the capillary^14^.

### Second harmonic generation

**Figure 6:**
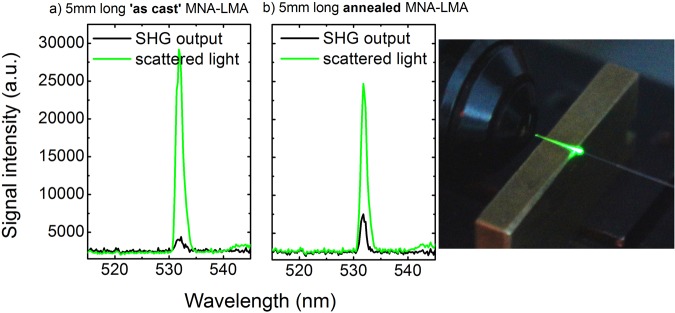
Second harmonic generation modal output (black line) and scattered light (green line) from 5 mm long samples: (**a**) MNA-LMA optical fiber (‘as-produced’) and (**b**) annealed MNA-LMA optical fiber. Right hand side picture: SHG from MNA-LMA fiber.

Finally, the MNA based MOFs were pumped for second harmonic generation using a 1064 nm Nd:YAG laser. Two composite optical fiber samples were examined: one ‘as-produced’ after capillary forces filling, and, one annealed using the procedure previously described. The results are presented in Fig. 6. For both optical fibers, scattered light (green lines in Fig. 6) is more intense than transmitted light at the fiber output, a fact that can be mainly attributed to the lack of phase matching. The large wavelength dispersion of MNA inherently introduces a considerable difference between the fundamental and second harmonic phase velocity, either for modes localized in the MNA-filled strands or those confined in the silica glass MOF core. Using equation^29^:

2 $${l}_{c}=\frac{\lambda }{2[{n}_{2\omega }-{n}_{\omega }]}$$

where *l*_c_ is the coherence length, *λ* and *n*_*ω*_ the vacuum wavelength and the refractive index of the fundamental mode, respectively, and *n*_*2ω*_ the refractive index of the second harmonic, one finds a coherence length of only *l*_c_ ≃ 4 μm for modes residing solely in the MNA filled strands. Focusing on the fundamental (and lower loss) ω and 2ω modes confined inside the MOF silica core, the corresponding coherence length manifold increases to ~84 μm. However, the overlap between the fundamental and the second harmonic mode in the fiber core is the dominant factor for conversion efficiency. Simulations preformed using the multi-pole method^30^ have shown that the specific modal overlap is limited; the 532 nm mode is of extended area as it is edging at the relevant PBG peak at 542 nm. Since in the current experiments the pump light was delivered using a 50 μm core silica optical fiber (multimode at 1064 nm), light output was unpolarized and hence SHG conversion was largely unoptimized. SHG output from the annealed fiber is almost double (85% more) compared to the ‘as cast’ fiber and the observed scattered light is 19% less for the same pumping conditions. As observed in the SEM images (see insets of Fig. 5), the more structured arrangement of the MNA crystals seems to increase SHG signal. This could be the result of the more structured crystal arrangement that augments SHG efficiency, and, secondly lower scattering losses due to improved crystallization, reducing leakage losses. Control of MNA crystallization inside the LMA capillaries and use of polarized pump light under the angle that meets the phase matching condition is expected to significantly enhance second harmonic conversion efficiency.

In summary, a new photonic crystal fiber was demonstrated by infiltrating an LMA-10 fiber with MNA, exhibiting PBG and MTIR guidance mechanisms and SHG light conversion properties. SHG is emitted at the slope of a bandgap formed in the optical fiber, namely that located at 542 nm, while excitation wavelength is in a bandgap-free zone, showing potential for future in-fiber photonic switches or optical limiting devices. Further studies are directed into the improvement of non-linear light conversion by applying thermal poling of the MNA material. We are also considering the use of the double guidance mechanism MOF design in light conversion for lasing and photovoltaic applications.

## Methods

The commercially available, all-silica, endlessly single mode LMA-10 drawn by NKT Photonics, was used as the host MOF for MNA infiltration. This MOF is comprised of a solid core surrounded by 4 rings of hexagonally arranged air-filled capillaries of radius r = 1.73 μm with a spacing between them of Λ = 7.2 μm. MNA in powder form and of 97% purity purchaced from Sigma-Aldrich, was used for the infiltration of the MOF capillaries without further purification. The LMA-10 optical fiber was immersed in an MNA melt pool resting at 140 °C (melting point of MNA ~131 °C) and left to be filled by capillary forces for 24 h. For the annealing experiments, the MOF under treatment was placed inside a temperature gradient from 110 °C to 50 °C and pulled out of it at a constant speed of 5 mm/hr.

**Figure 7:**
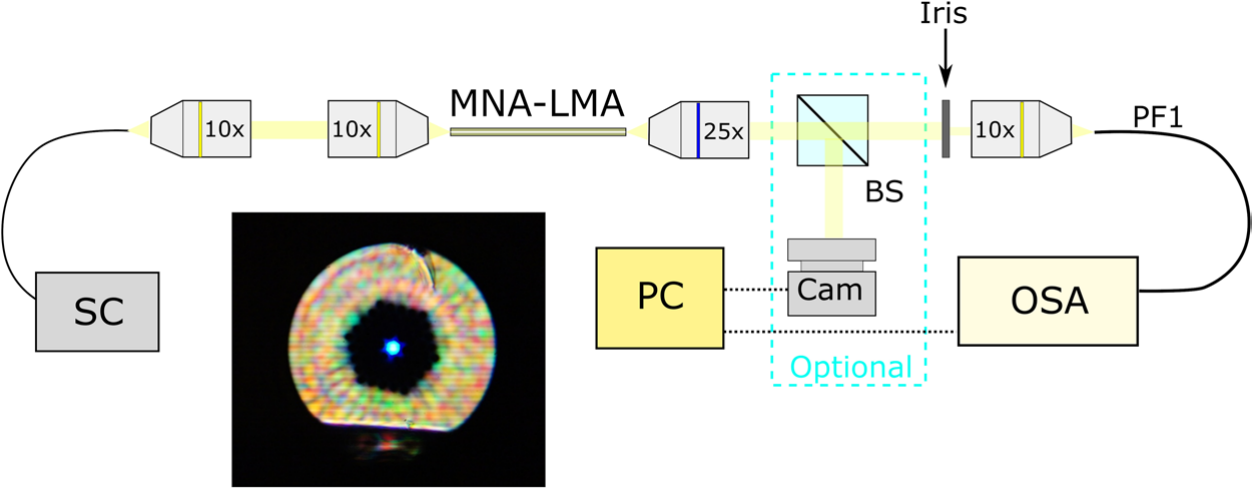
Experimental setup for the acquisition of the transmission spectra of the MNA-filled LMA-10 fiber. Inset (black box): Near field image of the MNA-LMA fiber output (without iris application for blocking the cladding light).

Spectral characterization was realized on the setup depicted in Fig. 7 comprised of a supercontinuum (SC) light source, objectives for light coupling/out-coupling, iris for blocking cladding light and a probe fiber of 50 μm core diameter (PF1) for measuring transmission and scattering spectra from the side of the fiber. Spectra were recorded using an optical spectrum analyzer (OSA). Optionally, a beamsplitter of 50:50 power split ratio was installed between the iris and the out-coupling objective lens to acquire near field images of the optical fiber output, using a digital camera (14MP APS-C sensor).

For second harmonic light generation a 155 ps Nd:YAG laser emitting at 1064 nm, at a repetition rate of 10 Hz, was used as pump source. The laser output was coupled into a 50 μm core (MM) silica optical fiber which acted as a beam homogenizer, in an endface, cladding pump arrangement. Typical energy per pulse at the MM optical fiber output was ~70 μJ, with the pulse broadened to ≳10 ns. Light was butt-coupled into 5 mm long MNA-LMA optical fibers using precision flexure stages without any polarization control. The 532 nm SHG light was collected using the probe fiber of an Ocean Optics HR4000 spectrometer and scattered light was collected from the composite fiber side at one fiber diameter distance, approximately.
